# Prenatal diagnosis and genetic counseling of a uniparental isodisomy of chromosome 8 with no phenotypic abnormalities

**DOI:** 10.1186/s13039-022-00594-1

**Published:** 2022-04-26

**Authors:** Chunjiao Yu, Ying Tian, Liang Qi, Bo Wang

**Affiliations:** 1 Department of Prenatal Diagnosis Center, Maternal, Child Health Hospital of Hubei Province, Wuhan, Hubei People’s Republic of China; 2 Department of Obstetrics, Maternal, Child Health Hospital of Hubei Province, Wuhan, Hubei People’s Republic of China; 3 Department of Ophthalmology, Maternal, Child Health Hospital of Hubei Province, Wuhan, Hubei China; 4 Department of Clinical Laboratory, Maternal, Child Health Hospital of Hubei Province, Wuhan, Hubei People’s Republic of China

**Keywords:** Uniparental disomy, Noninvasive prenatal testing, Chromosomal microarray analysis, Fluorescence in situ hybridization, Whole-exome sequencing

## Abstract

**Background:**

Uniparental disomy (UPD) refers to an epigenomic abnormality in which both copies of, or a part of, a homologous pair of chromosomes are inherited from one parent. UPD arises via a number of mechanisms, including monosomic and trisomic rescue (in embryonic development), incomplete segregation of chromosomes, and mitotic recombination.

**Case presentation:**

A 34-year-old, gravida 2, para 0 woman underwent amniocentesis at 18 weeks of gestation because the noninvasive prenatal testing (NIPT) showed the highly possibility of trisomy chromosome 8. GTG-banding karyotype analysis was performed on cultured amniocytes. Chromosomal microarray analysis (CMA), fluorescence in situ hybridization(FISH), whole-exome sequencing(WES) on uncultured amniocytes were performed.

**Results:**

CMA detected a 29.4 Mb uniparental isodisomy of chromosome 8, arr 8p23.3p12(168484_29427840) × 2 hmz [GRCh37(hg19)]. FISH, WES and ultrasound examination showed no abnormal. At the 36-month checkup, the baby was developing normally.

**Conclusion:**

Combination of NIPT,prenatal ultrasound, karyotype analysis, CMA, FISH, WES and genetic counseling will prove a more accurate risk assessment for the prenatal diagnosis of UPD.

## Introduction

Uniparental disomy (UPD) refers to an epigenomic abnormality in which both copies of, or a part of, a homologous pair of chromosomes are inherited from one parent [1]. UPD arises via a number of mechanisms, including monosomic and trisomic rescue (in embryonic development), incomplete segregation of chromosomes, and mitotic recombination [2].

UPD can be grouped into cases with pure isodisomy, pure heterodisomy and such with mixed iso-/heterodisomy. Also, one must distinguish UPD of a whole haploid chromosome set, UPD of one whole chromosome, and UPD of a part of a chromosome-a segmental UPD; the latter subtype is normally explained to be the consequence of a chromosomal rearrangement rescue-event [3]. UPD can be associated with human diseases through disruption of the normal allelic expression of genes that undergo genomic imprinting, homozygosity for an autosomal recessive trait, or due to incomplete (cryptic) trisomic rescue. Most often UPD is considered as a molecular genetic problem, but in some latest researches, it is affirmed and substantiated by corresponding data that UPD is a chromosomic disorder in the first place [4].

UPD does not affect the number or the structure of chromosomes and therefore escapes karyotype analysis [5], but it can be detected by SNP-based CMA technology, microsatellite analyses or trio exome sequencing (ES) [6]. Here we present the first case with paternal isodisomy 8 showing no phenotype [7].

## Methods

### Patients and samples

A 34-year-old, gravida 2, para 0 woman underwent amniocentesis at 18 weeks of gestation because NIPT showed the highly possibility of trisomy chromosome 8. GTG-banding karyotype analysis was performed on cultured amniocytes. CMA, FISH, WES on uncultured amniocytes were performed.

## Results

**Fig. 1:**
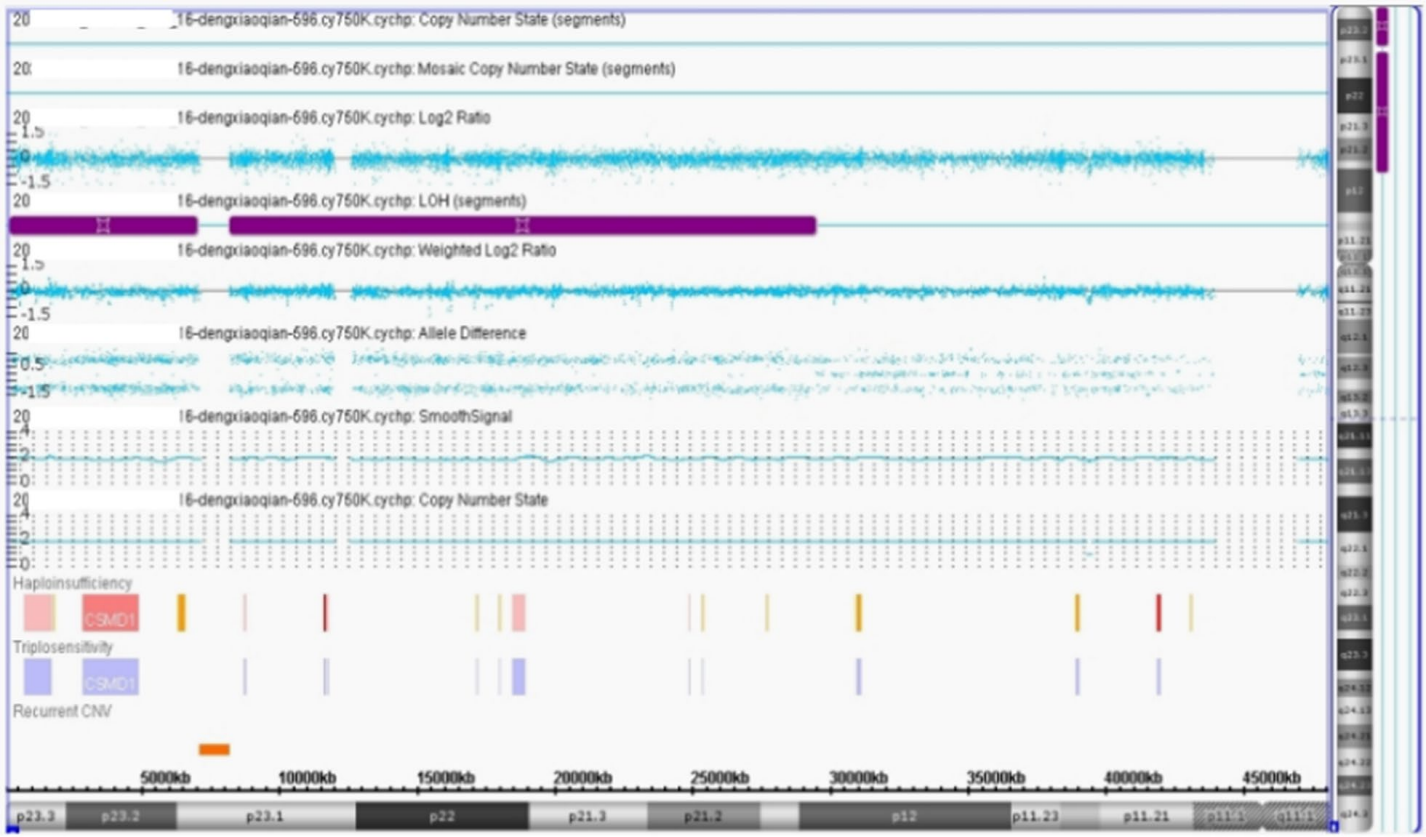
Uniparental isodisomy of chromosome 8, arr 8p23.3p12(168484_29427840) × 2 hmz [GRCh37(hg19)]

Cytogenetic analysis of the cultured amniocytes revealed a normal karyotype of 46,XX. CMA on uncultured amniocytes was performed using the Affymetrix CytoScan 750 K chip, which includes 550 k non-polymorphic markers and 200 k SNP markers. CMA detected a 29.4 Mb iso-UPD of chromosome 8, arr 8p23.3p12(168484_29427840) × 2 hmz [GRCh37(hg19)] (Fig. 1). The parental karyotypes and CMA analysis were normal. Microsatellite analyses suggested paternal UPD for chromosome 8.

All prenatal laboratory data were within normal range. Ultrasound examination showed no facial dysmorphisms or intrauterine growth restrictions (IUGRs) (At 25 weeks of gestation, estimated fetal weight700g, abdominal circumference 19.9 cm, head circumference 22.1 cm, femur long 4.3 cm and fetal heart rate 145 bpm)in the fetus [8]. After genetic counseling, we performed WES and FISH on uncultured amniocytes. The Novaseq6000 platform (Illumina, San Diego, USA), with 150 bp pair-end sequencing mode, was used for sequencing the genomic DNA of the family. The sequencing reads were aligned to the human reference genome (hg38/GRCh38) using the Burrows-Wheeler Aligner tool. WES revealed no homozygous mutations of any known recessive pathogenic genes for inherited disorders on chromosome 8p23.3p12. Interphase FISH analysis on uncultured amniocytes using the probe of CEP-8 revealed monosomy chromosome 8 in 5/200 cells and trisomy chromosome 8 in 3/200 cells.

The parents decided to continue the pregnancy. At 40 weeks of gestation, a 3350-g female infant was delivered naturally. Apgar scores were 8/9/9.The infant received a complete physical examination with normal findings. At the 36-month checkup, the baby was developing normally(head circumference 51 cm, height 99 cm, weight 14.5 kg, Intelligence Quotient, IQ = 115).

## Discussion

In contrast to numeric or structural chromosomal aberration, UPD does not affect the number or the structure of chromosomes and therefore escapes cytogenetic detection [5]. But it can be detected by SNP-based CMA technology and microsatellite analyses. SNP-based CMA consist of sets of oligonucleotides specific for polymorphisms in the genome. Although an oversimplification, each SNP has 2 different oligo sets, one for each allele, which when hybridized with sample DNA give a signal intensity relating to copy number and a SNP call referring to the allele in the sample, which can either be AA, BB, or the heterozygous call AB.

It must be stressed, that in SNP-based CMA only isodisomy can be detected and is normally blind for heterodisomy [7]. Microsatellite analyses or trio exome sequencing (ES) can detect pure isodisomy, pure heterodisomy, mixed iso-/heterodisomy and segmental UPD [6].

In this study, NIPT showed the highly possibility of trisomy chromosome 8. It is speculated that UPD of chromosome 8 may be caused by trisomy rescue.

UPD can be associated with human diseases through disruption of the normal allelic expression of genes that undergo genomic imprinting, homozygosity for an autosomal recessive trait, or mosaic aneuploidy. UPD could lead to various clinical phenotypes due to either homozygosity of recessive mutations or aberrant patterns of imprinting. Imprinting disorders alter epigenetic regulation and DNA methylation and histone modifications [9]. The widespread use of SNP-based CMA technology and microsatellite analyses have facilitated UPD detection. For the majority of chromosomes, UPD is without clinical consequence. However, for chromosomes 6, 7, 11, 14, 15, and 20, there are parent-of-origin or imprinting differences in gene expression in the context of UPD, which may lead to phenotypic abnormalities [10].

There are very few examples of paternal UPD8 in the literature, we present the first case with paternal isodisomy 8 showing no phenotype. We found some cases of paternal UPD8 with phenotypic abnormalities [7], the nosogenesis of most cases is the homozygote state of recessive pathogenic gene [11–16], but in other cases the cause is unknown [17]. There are several suspected recessive pathogenic genes for inherited disorders on chromosome 8p23.3p12 such as *XKR6, MIR597* [18]*, RP1L1* [19] and *PPP1R3B* [20]. In our case, no pathogenic mutations or homozygous recessive pathogenic genes were detected by CMA and WES.

## Conclusions

To summarize, we report a case of paternal UPD for chromosome 8 with a normal phenotype. Combination of NIPT,prenatal ultrasound, karyotype analysis, CMA, FISH, WES and genetic counseling will prove a more accurate risk assessment for the prenatal diagnosis of UPD.

## Data Availability

All relevant data and material is included in this publication.

## Abbreviations

UPD: Uniparental disomy
CMA: Chromosomal microarray analysis
NIPT: Noninvasive prenatal testing
FISH: Fluorescence in situ hybridization
WES: Whole-exome sequencing
IUGRS: Intrauterine growth restrictions
